# Calmodulin Supports TRPA1 Channel Association with Opioid Receptors and Glutamate NMDA Receptors in the Nervous Tissue

**DOI:** 10.3390/ijms22010229

**Published:** 2020-12-28

**Authors:** Elsa Cortés-Montero, María Rodríguez-Muñoz, M. Carmen Ruiz-Cantero, Enrique J. Cobos, Pilar Sánchez-Blázquez, Javier Garzón-Niño

**Affiliations:** 1 Department of Translational Neurosciences, Neuropharmacology, Cajal Institute, CSIC, Avenida Doctor Arce, 37. 28802 Madrid, Spain; elsa.cortes@cajal.csic.es (E.C.-M.); mrodriguez@cajal.csic.es (M.R.-M.); psb@cajal.csic.es (P.S.-B.); 2 Department of Pharmacology and Neurosciences Institute (Biomedical Research Center), University of Granada and Biosanitary Research Institute, ibs. Granada, 18016 Granada, Spain; rcmaric@ugr.es (M.C.R.-C.); ejcobos@ugr.es (E.J.C.)

**Keywords:** transient receptor potential A1, mu opioid receptor, calmodulin, glutamate NMDA receptor, neuropathic pain, inflammatory pain

## Abstract

Transient receptor potential ankyrin member 1 (TRPA1) belongs to the family of thermo TRP cation channels that detect harmful temperatures, acids and numerous chemical pollutants. TRPA1 is expressed in nervous tissue, where it participates in the genesis of nociceptive signals in response to noxious stimuli and mediates mechanical hyperalgesia and allodynia associated with different neuropathies. The glutamate N-methyl-d-aspartate receptor (NMDAR), which plays a relevant role in allodynia to mechanical stimuli, is connected via histidine triad nucleotide-binding protein 1 (HINT1) and type 1 sigma receptor (σ1R) to mu-opioid receptors (MORs), which mediate the most potent pain relief. Notably, neuropathic pain causes a reduction in MOR antinociceptive efficacy, which can be reversed by blocking spinal NMDARs and TRPA1 channels. Thus, we studied whether TRPA1 channels form complexes with MORs and NMDARs that may be implicated in the aforementioned nociceptive signals. Our data suggest that TRPA1 channels functionally associate with MORs, delta opioid receptors and NMDARs in the dorsal root ganglia, the spinal cord and brain areas. These associations were altered in response to pharmacological interventions and the induction of inflammatory and also neuropathic pain. The MOR-TRPA1 and NMDAR-TRPA1 associations do not require HINT1 or σ1R but appear to be mediated by calcium-activated calmodulin. Thus, TRPA1 channels may associate with NMDARs to promote ascending acute and chronic pain signals and to control MOR antinociception.

## 1. Introduction

TRPA1 is the only mammalian member of the transient receptor potential (TRP) ankyrin channel subfamily [1,2]. Four identical subunits assemble to form the functional TRPA1 nonselective cation channel permeable to Ca^2+^, Na^+^ and K^+^ [3]. The monomer contains six membrane-spanning domains and a presumed pore-forming region between the fifth and sixth transmembrane domains. The long N-terminal segment, which contains at least 16 ankyrin repeat domains as the shorter C-terminal region are predicted to be in the cytoplasm [4,5]. TRPA1 was initially suggested to function as a thermodetector of noxious cold stimuli [6]. Although this hypothesis remains controversial [7], there is widespread agreement that TRPA1 plays an important role in chemonociception. TRPA1 detects exogenous irritant compounds (e.g., formalin, acrolein, tear gas, chlorine, isothiocyanates and thiosulfinates from mustard, cinnamon, wasabi and garlic) and endogenous compounds (e.g., reactive oxygen species, bradykinin and 4-hydroxynonenal) produced during tissue damage or neurogenic inflammation [8,9,10,11]. Additionally, TRPA1 is activated by non-electrophilic molecules such as menthol and cannabinoids [12,13].

Painful peripheral stimuli are initially detected by primary afferent sensory nerve fibers (C and Aδ fibers) that have their soma in the trigeminal ganglia and the dorsal root ganglia (DRG). These peripheral neurons transmit this information to neurons within the spinal cord and thence to the brain via ascending neural circuits [8]. One of the most important sensory transducers that detects and transmits noxious stimuli is the transient receptor potential (TRP) ion channel family [11]. TRPA1 is expressed by nociceptive neurons of DRG and trigeminal ganglia, as well as in cutaneous primary afferent terminals [14]. *Trpa1* mRNA has been localized to small- and medium-diameter sensory neurons, many of which are C- and Aδ-fiber nociceptors [12,15,16]. In the spinal dorsal horn, TRPA1 ion channels on the central terminals of peptidergic primary afferent nerve fibers regulate transmission to glutamatergic and GABAergic interneurons [17,18]. TRPA1 has been presented as a candidate to mediate inflammatory mechanical hyperalgesia as well as cold hyperalgesia under inflammatory conditions; indeed HC-030031, a TRPA1 selective antagonist, attenuates inflammatory- and neuropathy-induced mechanical hypersensitivity [19,20]. TRPA1 channels are also involved in the maintenance of mechanical and cold hyperalgesia in persistent inflammation [20]. Furthermore, formalin excites sensory neurons by directly activating TRPA1. In fact, blockade of TRPA1 in vivo, either using a specific antagonist or through disruption of the TRPA1 gene substantially attenuates pain-related responses to formalin [21]. Thus, based on its localization and functional properties, TRPA1 is considered a key player in acute and chronic (neuropathic) pain [22,23,24,25].

On the other hand, a series of neural changes associated with neuropathic pain originate from anomalous persistent activation of N-methyl-d-aspartate glutamatergic receptors (NMDARs), which triggers a cascade of events leading to central nervous system hypersensitization (“central windup”) [26,27]. In fact, neuropathic pain characterized by tactile allodynia and hyperalgesia remits after blockade of NMDAR function [28]. NMDARs in the spinal dorsal horn play a key role in chronic pain and central sensitization [29] and their interaction with opioid receptors plays an important role in regulating nociceptive transmission between primary afferents and dorsal horn neurons. NMDARs are present in primary afferents [30,31] and evoke the release of substance P from their central terminals [32]. Conversely, substance P release from primary afferents is inhibited by mu-opioid receptors (MORs) [33,34] and delta-opioid receptors (DORs) [35]. In the latent sensitization model of chronic pain, spinal cord hyperalgesia is maintained by NMDARs [36] and suppressed by MORs, DORs and kappa-opioid receptors [37]. In nervous tissue, NMDARs play an essential role in MOR desensitization [38,39] and neuropathic pain leads to reduced morphine efficacy and more rapid development of tolerance [40,41]. Interestingly, antinociceptive morphine tolerance is attenuated by blocking spinal TRPA1. This suggests that spinal TRPA1 may also contribute, at least in part, to the facilitation of morphine antinociceptive tolerance [42].

MOR, via histidine triad nucleotide-binding protein 1 (HINT1) and type 1 sigma receptor (σ1R), connects with the NR1 subunit of NMDARs [43,44] and positively regulates NMDAR calcium (Ca^2+)^ fluxes [45]. σ1R binds to the NMDAR NR1 subunit in a Ca^2+^-dependent manner [44,46] and thus prevents the binding of the NMDAR negative regulator calmodulin (CaM) [47,48,49]. Notably, the HINT1 protein and CaM bind to the N- and C-terminal domains of various TRPs, including TRPA1. σ1R competes with CaM for binding to TRPs, except for its binding to the TRPA1 C-terminus, where σ1R promotes binding of CaM [50].

The aforementioned evidence prompted us to investigate whether TRPA1 channels associate with MORs, DORs and NMDARs. The study was performed principally on DRG, spinal cord and cortex and the possibility of direct associations was explored in in vitro assays with cloned sequences of these proteins. Ex vivo studies indicated the association of TRPA1 with MORs, DORs and NMDARs in vivo and these associations were altered by pharmacological interventions, the administration of a chemical irritant such as formalin and during neuropathic pain. The in vitro assays suggest a role for CaM in the association of TRPA1 with MORs and the NR1 regulatory subunit of NMDARs.

## 2. Results

### 2.1. TRPA1 Channel Association with MORs, DORs and NR1 Subunits, in the Cerebral Cortex, Spinal Cord and DRGs. Effect of Pharmacological Interventions

**Figure 1:**
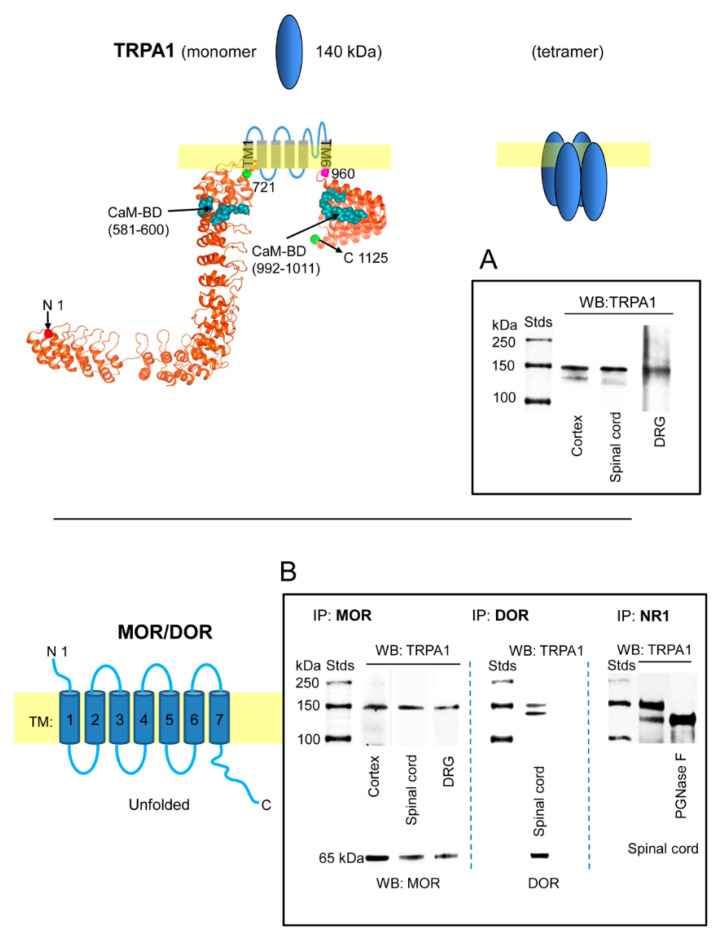
Expression of transient receptor potential ankyrin member 1 (TRPA1) channels in the cerebral cortex, spinal cord and dorsal root ganglia (DRG). TRPA1 association with mu-opioid receptors (MORs), delta-opioid receptors (DORs) and NR1 subunits. The structural model of the TRPA1 monomer was predicted by Novafold (DNASTAR Inc., Madison, WI, USA). The N- and C-terminal cytosolic sequences are linked by six transmembrane domains (TM). Ribbon model: The 3D structure of N- and C-terminal sequences shows the CaM-binding domains (BD) indicated by blue spheres. (**A**) Immunodetection of the TRPA1 monomer in synaptosomal fractions of murine cerebral cortex, spinal cord and dorsal root ganglia (DRG). (**B**) The membrane preparations were solubilized and the MOR, DOR and NR1 subunits of glutamate NMDARs were immunoprecipitated. The coprecipitation of the TRPA1 monomer was addressed by Western blotting. The expected size of the TRPA1 monomer is approximately 140 kDa; however, the immunosignals usually form a doublet of 140–150 kDa. The material associated with NR1 subunits was subjected to deglycosylation with PGNase F, which abolished the TRPA1 150 kDa band in favor of the 140 kDa band. Further details in Section 4.

We confirmed the presence of TRPA1 channels in DRG, spinal cord and cerebral cortex of murine nervous tissue [4,16,25,51,52,53]. The 1125 residue monomer was immunodetected in synaptosome-enriched fractions using affinity-purified antibodies against the N-terminal region and an extracellular sequence. The opioid receptors MOR and DOR are present in the cortex and dorsal horn (substantia gelatinosa) of the spinal cord; they are also expressed on cell bodies of the sensory neurons (DRG) and are transported to their central terminals in the superficial dorsal horn and to peripheral terminals in peripheral tissues [54,55]. We observed that immunoprecipitated MOR and DOR were accompanied by TRPA1-related signals (Figure 1B), suggesting that they form functional complexes in nervous tissue. Similarly, the NR1 subunit of NMDARs coprecipitated TRPA1 in the spinal cord. The immunosignals mostly appeared as a doublet at approximately 140 and 150 kDa (Figure 1A), which is probably due to the described pair of N-glycosylation sites in the external loop of human TRPA1, which is flanked by the transmembrane regions TM1 and TM2 (721–960) [56]. Computer analysis indicated this possibility for murine TRPA1 [57] and in vitro, PGNase F abolished the 150 kDa band augmenting the intensity of the expected 140 kDa (Figure 1B).

**Figure 2:**
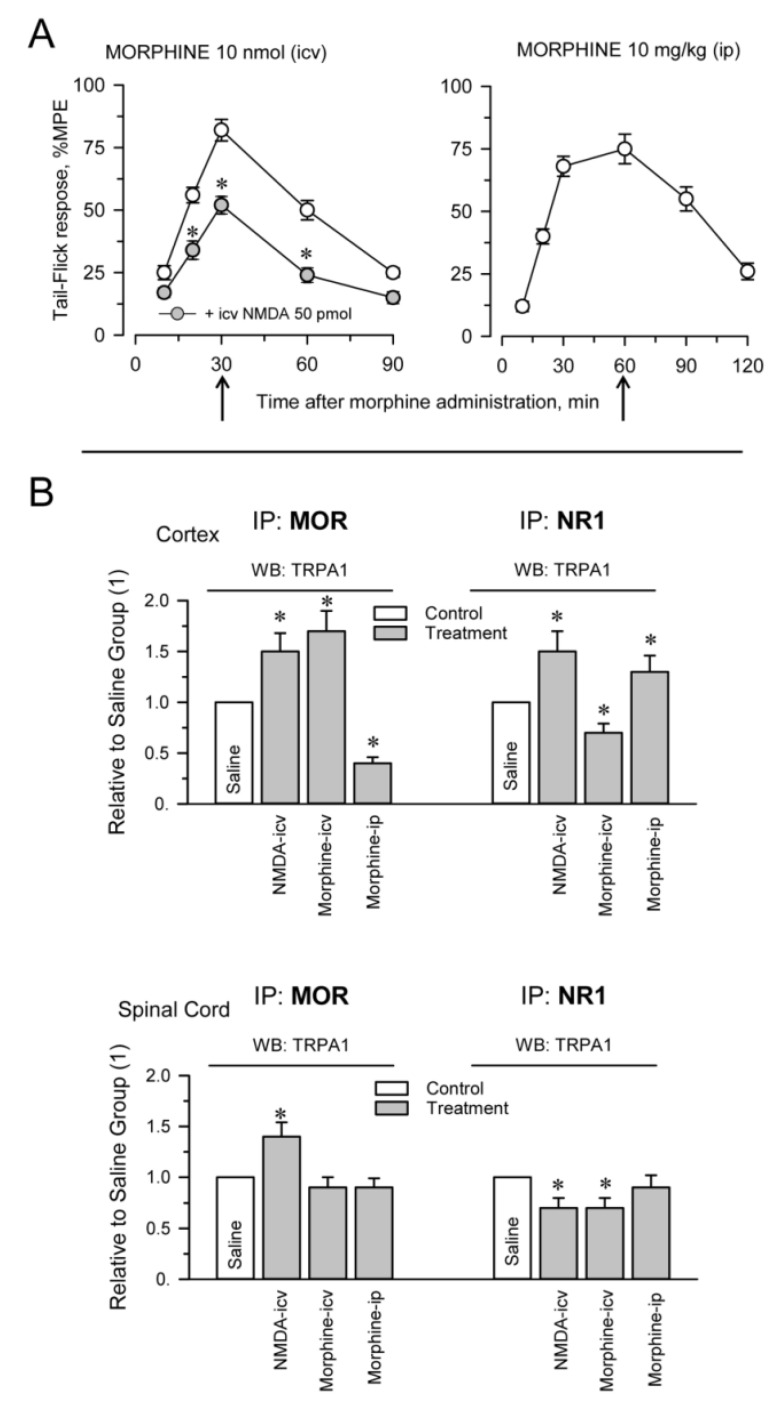
Pharmacological modulation of TRPA1 associations with MORs and glutamate NMDARs. Different groups of mice were icv-injected with saline, 50 pmol NMDA and 10 nmol morphine. Another group of mice received 10 mg/kg morphine via the ip route. (**A**) Antinociception promoted by morphine was determined in the warm water tail-flick test and is shown as a time-course. The effect of NMDA is shown relative to its antagonism of icv morphine analgesia. The points are the mean ± SEM from groups of six mice. * Significant differences with respect to the group that received only morphine icv, *p* < 0.05. (**B**) Effect of pharmacological interventions on the associations of TRPA1 with MORs and NR1 subunits. Thirty minutes after the icv injections and 60 min after ip morphine (indicated by the arrows), the mice were killed and cortical and spinal cord synaptosomal fractions were prepared. Following solubilization of these membrane preparations, MORs and NR1 subunits were immunoprecipitated. The coprecipitated TRPA1 was detached from the bait proteins and the presence of the monomer was analyzed by Western blotting. For each association and structure, namely, the cerebral cortex and spinal cord, the data were referred to the control group that received saline and was assigned an arbitrary value of 1. The bars are the mean ± SEM from two or three determinations. * Significant differences with respect to the control group, *p* < 0.05. Further details in Supplementary Materials Figure S4 and Section 4.

The plasticity of protein complexes in response to pharmacological interventions and/or physiological alterations usually indicates their functional relevance. This possibility was addressed by injecting morphine, MOR agonist, via intracerebroventricular (icv; first or second ventricles) and intraperitoneal (ip) routes and NMDA, an activator of glutamate NMDARs, via the icv route. Morphine, at the dosage used, injected via the icv or ip route promoted similar levels of antinociception, approximately 75% of the maximum measurable effect in the analgesic test. The effect of the opioid peaked sharply at 30 min after icv injection, while antinociception was more sustained, with a maximum between 30 and 60 min, after ip administration. On the other hand, icv NMDA diminished the antinociception produced by supraspinal icv morphine (Figure 2A). We observed that icv-injection of NMDA increased MOR-TRPA1 and NR1-TRPA1 associations at the cortical level and the spinal cord level. On the other hand, icv morphine increased ex vivo coprecipitation of MOR-TRPA1 complexes at the cortex level and diminished that of NR1-TRPA1 at the two levels of the nervous system studied. Morphine injected via the ip route mostly modified both associations at the cortical level (Figure 2B).

### 2.2. Inflammatory and Neuropathic Pain Alters TRPA1 Channel Association with MORs and NMDARs

**Figure 3:**
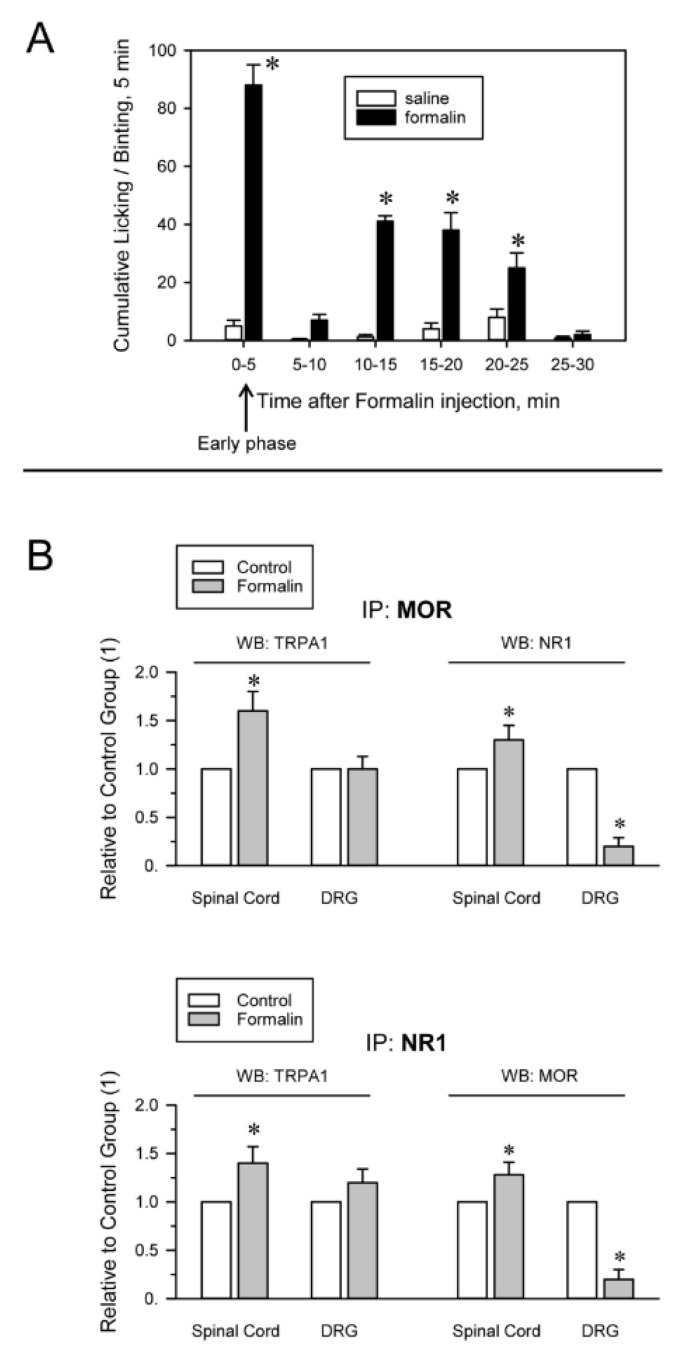
Formalin-induced inflammatory pain alters TRPA1 associations with MORs and NMDARs. (**A**) The mice received at time 0 an intraplantar injection of saline (control group) or 20 μL of a 0.5% formalin solution into one of the hind paws. The time spent licking/biting was recorded every 5 min by a blinded observer. The bars are the mean ± SEM from three determinations. * Indicates significant difference compared to the saline control group, *p* < 0.05. (**B**) The animals were sacrificed 5 min after the injection, when the early phase of formalin-induced pain had already peaked (indicated by the arrow). DRG and spinal cords were obtained. MORs and NR1 subunits were immunoprecipitated from the solubilized membrane preparations. At the end of the procedure, coprecipitated TRPA1 was detached from the bait proteins and analyzed by Western blotting * Indicates significant difference compared to the saline control group, *p* < 0.05. Further details in Figure 2, Supplementary Materials Figure S5 and Section 4.

The injection of formalin into the hind paw is widely used to evaluate the effects of analgesic compounds in laboratory animals. Intraplantar formalin excites sensory neurons by activating excitatory TRPA1 channels and this activity underlies the physiological and behavioral responses associated with this model of pain hypersensitivity, such as licking and biting responses [21,58]. The intraplantar injection of 0.5% formalin produced a biphasic behavioral reaction, with an initial phase lasting 5 min and a quiescent period, followed by a second less intense phase lasting from 10 to 25 min. Mice receiving saline, a solvent of formalin, did not show appreciable nociceptive-related responses during the time interval evaluated (Figure 3A). The mice did not exhibit pain-like behaviors toward the noninjected hind paw. We analyzed whether activation of TRPA1 channels on peripheral nerve terminals influences their association with MORs and NMDARs as well as the MOR-NR1 association in the spinal cord and DRG. Formalin almost completely abolished the MOR-NR1 association in DRG but increased this association in the spinal cord. The association of TRPA1 with MOR and NR1 subunits increased in the spinal cord and TRPA1-NR1 showed a tendency to augment in DRG (Figure 3B).

**Figure 4:**
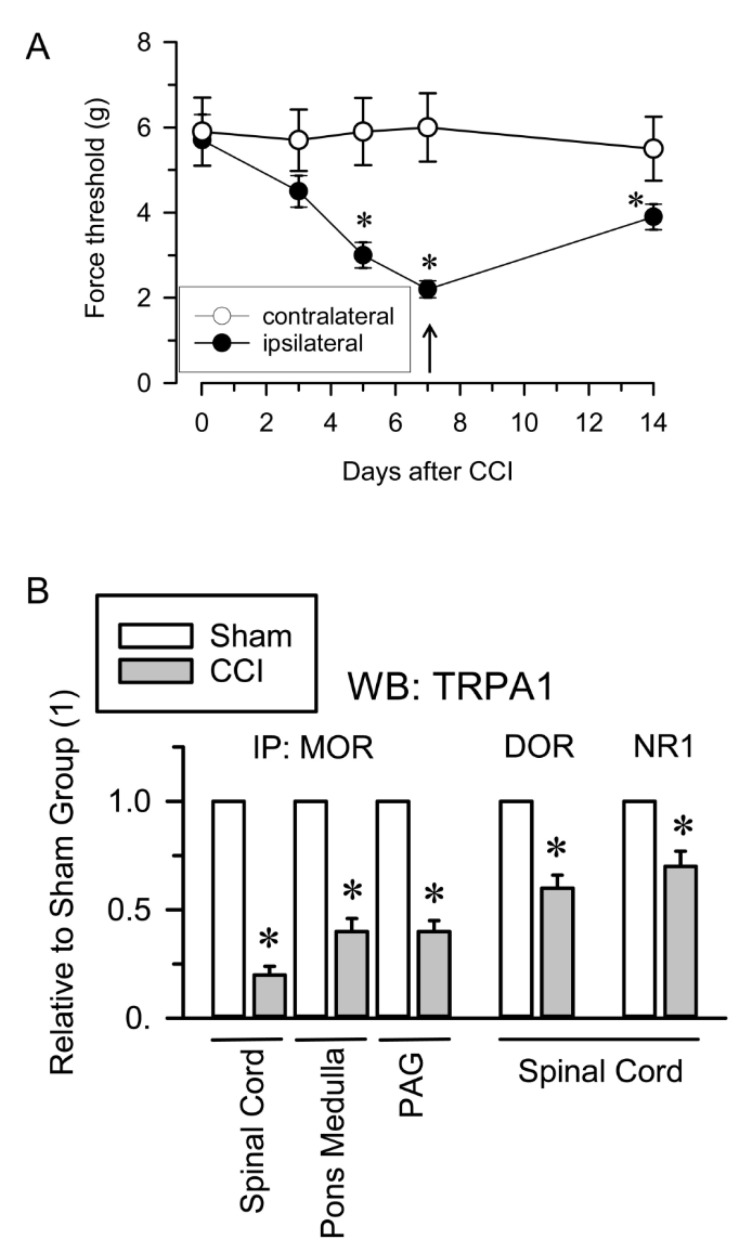
TRPA1 associations with opioid receptors and NMDARs in the CCI model of neuropathic pain. (**A**) Induction of mechanical allodynia. Chronic constriction injury (CCI) of the sciatic nerve caused neuropathic pain in mice. The paw withdrawal thresholds of the contralateral and ipsilateral paws of the mice were measured before (indicated as 0) and 3, 5, 7 and 14 days after surgery. The force (in grams) at which the mice withdrew their paws in response to von Frey hair stimulation was determined as an index of mechanical allodynia. The data are the mean ± SEM of six mice. * Indicates significant difference compared to the nociceptive threshold of the sham-operated control group on day 0 (7th after surgery); *p* < 0.05. (**B**) Seven days after surgery, sham and CCI mice were killed and synaptosomal fractions from different neural areas were obtained. The MOR, DOR and NR1 subunits were immunoprecipitated from the solubilized membrane preparations and coprecipitated TRPA1 was analyzed by Western blotting. * Indicates significant difference compared to the sham control group, *p* < 0.05. Further details in Figure 2, Supplementary Materials Figure S6 and Section 4.

The influence of neuropathic pain on the association of TRPA1 channels with opioid receptors and NMDARs was also evaluated. Neuropathic pain was induced by chronic sciatic nerve constriction injury (CCI). The nerve-injured mice maintained a healthy appearance and were well groomed and their body weight decreased after surgery but returned to preoperative values within 2–4 days. Seven days after surgery, CCI mice but not sham-operated mice showed allodynia and on days 12 to 15, the nociceptive responses of CCI mice nearly returned to pre-surgery levels [59] (Figure 4A). The Sham and CCI mice were sacrificed on day 7 and the nervous structures were obtained for immunoprecipitation studies. Since cortical NMDAR-MORs associations remain unchanged in the murine CCI model of neuropathic pain [41], we analyzed the extent of their interaction in the pons-medulla and periaqueductal gray matter (PAG). We observed reductions in the MOR-TRPA1 association of ≥50% in the pons-medulla, PAG and spinal cord. Similarly, the DOR-TRPA1 and NR1-TRPA1 associations were significantly diminished in the spinal cord of CCI mice (Figure 4B).

Because NMDARs connect with MORs via HINT1 proteins and σ1Rs [46,60], we explored this possibility for the associations of TRPA1 channels with MORs and NMDARs. We evaluated this possibility in P2 fractions from the spinal cords of HINT1^−/−^ and σ1R^−/−^ mice. In these mice, the associations of TRPA1 with these proteins were comparable to those of the corresponding wild-type mice (Supplementary Materials Figure S1).

### 2.3. Calmodulin Mediates TRPA1 Channel Association with MORs and NR1 Subunits of Glutamate NMDARs

**Figure 5:**
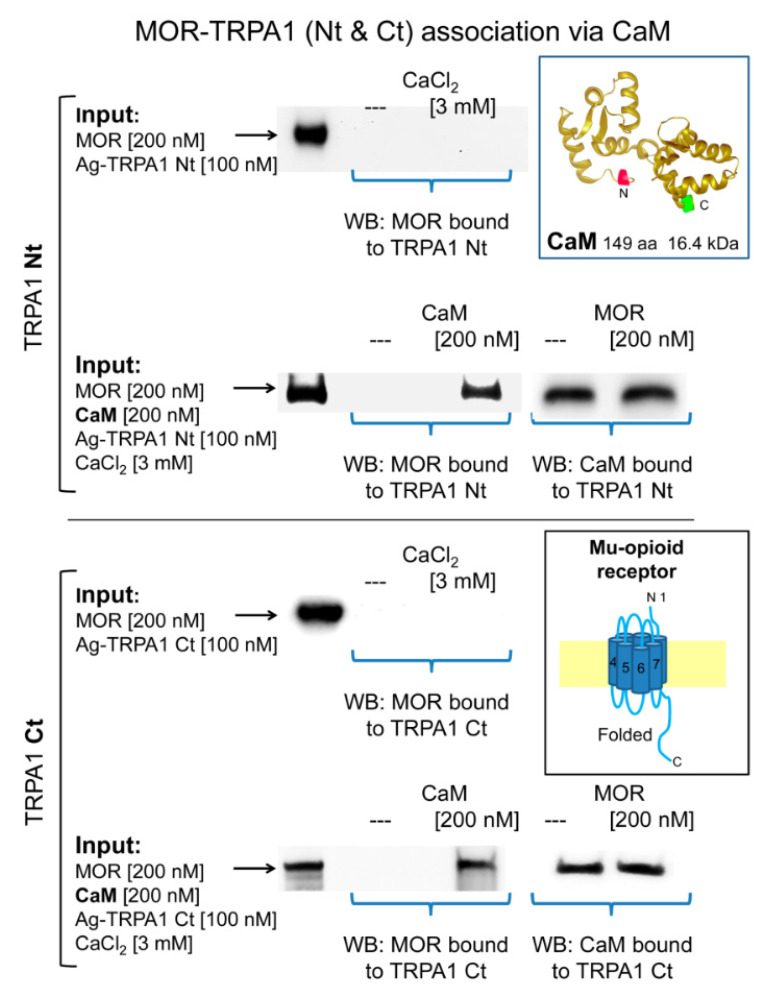
Calmodulin mediates the association of TRPA1 with MORs. The recombinant cytosolic Nt and Ct regions of TRPA1 were covalently attached to N-Hydroxysuccinimide (NHS)-agarose beads and then incubated with MOR in the absence and presence of 3 mM CaCl_2_. In another set of assays, the TRPA1 regions were sequentially incubated with calmodulin (CaM) and MORs in the presence of 3 mM CaCl_2_. At the end of the incubation, agarose-TRPA1 was recovered by several cycles of washing-resuspension and the bound proteins were detached with 2× Laemmli buffer and resolved by SDS-PAGE followed by immunodetection. Further details in the Methods. Key: Input, Proteins incubated with agarose TRPA1, MORs are immunodetected; WB, immunodetected protein in Western blot.

**Figure 6:**
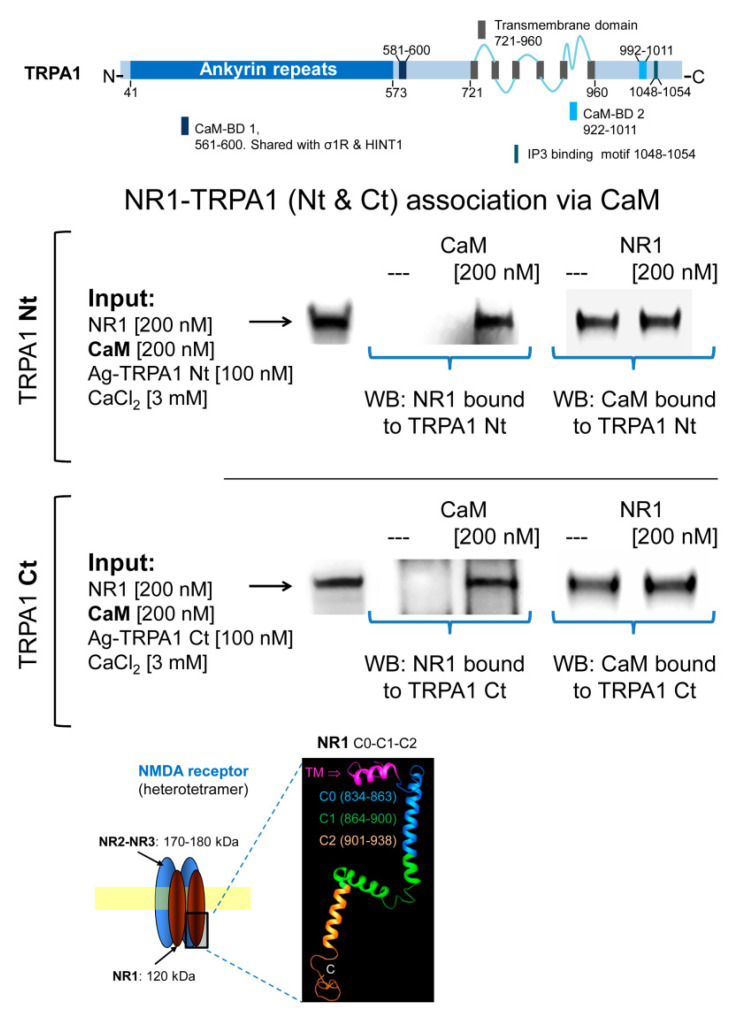
Calmodulin mediates the association of TRPA1 with NMDARs. Linear representation of TRPA1 monomer showing CaM- and IP3-binding domains (BD) [5]. Recombinant cytosolic Nt and Ct regions of TRPA1 were covalently attached to NHS-agarose beads and then were sequentially incubated with CaM and NR1 subunits of NMDARs in the presence of 3 mM CaCl_2_ and 30 µM of a peptide mapping the C1 region of NR1 subunit (879–888: TFRAITSTLA) [46]. CaM and NR1 associated with TRPA1 regions were detected by Western blotting. Details in Figure 5 and Section 4.

In in vitro assays, we observed that MOR does not bind to cytosolic TRPA1 Nt or Ct regions and that 3 mM CaCl_2_ does not promote this association (Figure 5). In this paradigm, σ1R failed to couple TRPA1 Nt or Ct to the MOR and the HINT1 protein also failed to bride MOR to TRPA1 Nt (Supplementary Materials Figure S2). Because HINT1 does not bind to TRPA1 Ct, this assay was not extended to this region [50]. However, in the presence of Ca^2+^-activated CaM, TRPA1 Nt and Ct coupled to MORs (Figure 5). CaM binds to TRPA1 Ct in the absence of Ca^2+^ and this association increases at a physiological 3 mM concentration of Ca^2+^ [50]. Notably, the MOR-CaM interaction showed a similar pattern of dependence on Ca^2+^ and the MOR-TRPA1 Ct association was observed even in the absence of Ca^2+^ (Supplementary Materials Figure S3). The NR2A and NR2B subunits of NMDARs form stable associations with regulatory α2δ1 proteins [61], which may block the access of these subunits to TRPA1 channels. Thus, we evaluated the possibility that the cytosolic region of the NR1 subunit interacting with TRPA1 monomers. We observed that NR1 does not interact directly with TRPA1 Nt or Ct but in the presence of 3 mM Ca^2+^, CaM facilitated these interactions (Figure 6).

## 3. Discussion

This study suggests that a relationship exists between TRPA1 and glutamate NMDARs. Both cationic channels participate in the genesis of noxious signals, are functionally coupled to opioid receptors such as the MOR and form associations, which are modulated by their activity.

The TRPA1 channel was first cloned from human fetal lung fibroblasts and is also expressed in other types of nonneuronal cells, including keratinocytes, melanocytes, mast cells, odontoblasts, enterochromaffin cells and β-cells of the Langerhans islets [62,63,64]. In murine peripheral sensory neurons, TRPA1 is expressed in peptidergic (TRPV1+) neurons [4]. In the spinal dorsal horn, TRPA1 on the central terminals of peptidergic primary afferent nerve fibers regulates transmission to glutamatergic and GABAergic interneurons [12,15,16]. In the central nervous system, TRPA1 is also present in the somatosensory cortex, hippocampus, pons-medulla and spinal cord [52,53,65,66].

Our study identified the presence of TRPA1 channels in the synaptosomal fraction of murine frontal cortex, PAG, pons-medulla, spinal cord and DRG, where MORs and DORs are also expressed. The finding that TRPA1 and MOR/DOR coprecipitate suggests a functional relationship between the nociceptor channel and these antinociceptive G protein-coupled receptors (GPCRs). Glutamate NMDARs also associate with MORs via NR1 subunits of NMDARs that carry the regulatory cytosolic C1 segment [43]. This association is regulated by HINT1 proteins and σ1Rs, with the latter competing with the NMDAR inhibitor CaM [47] for binding to NR1 subunits [44,46]. The activation of MORs recruits the activity of NMDARs, which exerts negative feedback on MOR signaling. This mechanism mostly operates for opioids such as morphine, which promotes limited internalization of MORs and mediates the short-term tolerance that follows the effects of high doses of opioids [48].

In the resting state, the association of MORs with NMDARs elevates the threshold for direct NMDAR activation and facilitates the recruitment of this glutamatergic activity in response to MOR signaling [43,48,60]. The MOR-NMDAR association is supported by HINT1 and σ1Rs and accordingly, in mice lacking expression of these proteins, the MOR-NMDAR association greatly diminishes [44,67]. Our data indicate that TRPA1 does not couple to MORs or NMDARs via HINT1 or the σ1R and thus the TRPA1-MOR and TRPA1-NR1 associations were maintained in neural P2 fractions obtained from HINT1^−/−^ and σ1R^−/−^ mice. In in vitro assays, the HINT1 protein or σ1R did not promote such associations but in the presence of CaM, the Nt and Ct cytosolic regions of TRPA1 coupled with the MOR and the NR1 subunit. The TRPA1 monomer binds HINT1, σ1R and CaM via the N-terminal region and σ1R and CaM but not HINT1 via the C-terminal region. Calcium promotes CaM and σ1R binding to TRPA1 Nt and Ct but CaM also binds to TRPA1 Ct in the absence of Ca^2+^. Moreover, σ1R competes with CaM at the TRPA1 Nt but promotes CaM binding to the Ct region [50]. The regulation of TRPA1 activity by CaM appears to be complex and thus, CaM mediates Ca^2+^-dependent potentiation and inactivation of the channel. TRPA1 contains a noncanonical CaM binding domain (CaMBD) in the C-terminal region, while the N-terminal region, which exhibits weak CaM binding, is critical for Ca^2+^-induced desensitization [68,69]. The CaMBD in the N-terminus is close to the linker domain and/or pre-S1 helix, regions structurally near the C-terminal CaMBD [5], which is consistent with previous studies demonstrating that extracellular Ca^2+^ regulates TRPA1 by acting through a channel site that needs to be very close to the channel pore [16]. Thus, the topology of the CaMBDs on TRPA1 makes the simultaneous interaction of CaM with the TRPA1 channel and cytosolic regions of the MOR and NR1 subunits of NMDARs feasible.

At low activation levels, MORs couple with NMDARs and TRPA1 channels and the activation of MORs promoted by icv morphine diminishes the MOR-NMDAR association [46] but enhances the MOR-TRPA1 association at the supraspinal level. Under low Ca^2+^ influx, TRPA1 Ct and MORs bind to CaM and thus, these signaling proteins may form basal complexes. The activation of TRPA1 channels, for example, by formalin, may augment CaM binding; hence, the MOR-TRPA1 association will increase, as observed for the spinal cord. When the MOR is activated, the interplay between σ1R and CaM at the TRPA1 Nt will diminish CaM binding and thus the MOR association but at the TRPA1 Ct, σ1R always promotes CaM binding, which supports MOR association. After icv morphine, the latter situation was observed at the cortical level but not at the spinal level, probably because icv-injected morphine barely reaches spinal MORs. On the other hand, ip-injected morphine produced the opposite effect, diminishing the MOR-TRPA1 association at the cortex but not at spinal level. Morphine, when injected by the systemic route, crosses the blood brain barrier but mostly acts at the peripheral and spinal levels [70]. The opioid receptors, MOR and DOR, are present in the dorsal horn (substantia gelatinosa) of the spinal cord, cell bodies of sensory neurons (DRG), central terminals in the superficial dorsal horn and peripheral terminals in peripheral tissues [54,55]. Although, ip morphine did not alter TRPA1-MOR association in the spinal cord, the reduction in these complexes observed in the cortex may originate at the MORs located in sensory neurons reaching the upper areas through the neuromodulation of ascending pathways.

MOR-activated NMDARs participate in a negative feedback loop that dampens MOR signaling; however, there is no evidence that activated TRPA1 channels promote such an effect. On the other hand, GPCRs such as type 1 mGluR5 increase TRPA1 sensitivity and this regulation requires the activity of PKA and PKC on specific residues of the channel monomer [71]. Similarly, in DRG, bradykinin B2 and the PAR2 receptor can also stimulate TRPA1 function, probably via Gq/G11-PLC-PKC [51,72]. The TRPA1 residues identified as targets for PKA, PKC and Cdk5 phosphorylation are outside the CaMBDs [71,73,74] and thus should not interfere with CaM binding or CaM-mediated associations of the channel with additional partner proteins. In this scenario, MORs, by inhibiting sensory neuron excitability [75], may oppose TRPA1 channels; however, MOR activity may also contribute to TRPA1 activity via PKC, PKA and Cdk5 [48]. Indeed, the MOR can contribute zinc ions and nitric oxide (NO) to the regulation of TRPA1 channels. The C-terminal region of the MOR couples via HINT1 proteins to regulators of GTPase signaling (RGS) proteins 20 and 17 (RGSZ1 and RGSZ2), which carry zinc ions bound to cysteine rich domains (CRDs). In the MOR-mediated complex, the N-terminal region of neural nitric oxide synthase (nNOS) binds to these RGSZ proteins. Upon activation of MORs, nNOS generates NO and releases zinc ions from RGSZ CRDs [48,49]. Because, intracellular zinc ions activate TRPA1 through interactions with Cys641 and Cys1021 in the C-terminal fragment [76], MORs may increase TRPA1 activity, providing zinc ions but it may also reduce its activity by removing Cys-bound zinc ions via NO.

NMDARs are present on nociceptive primary afferents from the skin [77,78,79] and trigeminal ganglia [80]. Approximately 47% of unmyelinated peripheral axons are immunopositive for NMDARs and are believed to be involved in synaptic transmission between primary afferents and dorsal horn neurons [81,82]. NMDARs play a relevant role in central sensitization in spinal neurons and are also involved in the release of specific neurotransmitters during pain transmission at both the peripheral and central terminals of sensory neurons [83]. Thus, NMDA receptors may be involved in functionally divergent nociceptive systems [84]. The activation of MORs or the direct activation of NMDARs by icv NMDA brings about reductions in the MOR-NR1 association [43]. These situations recruit protein kinases such as PKC, PKA and CaMKII [48], which, via the phosphorylation of MOR cytosolic residues, exert negative feedback on opioid signaling and disrupt the MOR association with NMDARs. The binding of Ca^2+^-CaM to NR1 reduces the probability of NMDAR channel opening, while phosphorylation of the NR1 cytosolic region by PKC and PKA blocks access to CaM, increases the binding of σ1R and promotes NMDAR function [46,47].

In contrast, the interaction of the TRPA1 channel with MORs and NMDARs relies on CaM. Supraspinal administration of NMDA increased the MOR-TRPA1 association at the cortical and spinal levels and promoted or diminished NMDAR-TRPA1 associations at the cortical and spinal level, respectively. NMDA-activated NMDARs increase cytosolic Ca^2+^ and thus, may augment the binding of supraspinal TRPA1 channels with the aforementioned proteins via Ca^2+^-activated CaM. This mechanism may trigger the changes observed at the spinal level via modulation of descending pathways, which finally affect TRPA1 association with MORs and NMDARs in the opposite direction. Formalin also brought about increases in TRPA1 association with MORs and NMDARs, particularly in the spinal cord and caused a great reduction in the MOR-NMDAR association in DRG, with values comparable to those produced by CCI in the spinal cord, pons-medulla and PAG [41]. These changes may be mediated by the activity of NMDARs and indeed, NMDAR antagonists attenuate formalin-induced pain behaviors [84], suggesting that the glutamatergic receptor is necessary for TRPA1 nociceptive signals to propagate. The icv or ip activation of MORs also affected the association between TRPA1 and NMDARs, probably by recruiting the activity of NMDARs [43,46]. Morphine icv, reduced cortical and spinal TRPA1-NMDAR associations and ip morphine increased this association at the cortical level.

The associations of TRPA1 with these signaling proteins show a more uniform change profile as a consequence of neuropathic pain. Mechanical allodynia caused by CCI reduced the association of TRPA1 with MOR, DOR and NMDARs. Disruption of the MOR-NMDAR association is also observed in this animal model of neuropathy [41]. Thus, adaptive responses to the sustained overactivity of NMDARs “winding up” differ from those underlying acute pharmacological interventions and inflammatory formalin-induced pain. In summary, our study has shown the association of TRPA1 channels with MOR, DOR and NMDARs in different areas of the nervous system, as well as the plasticity of such associations in response to drugs and inflammatory and neuropathic pain. These findings may provide new avenues for understanding TRPA1 physiology in relation to signaling proteins relevant to pain perception.

## 4. Materials and Methods

### 4.1. Animal and Drugs

Male albino CD1 mice (ENVIGO, Horst, The Netherlands), homozygous (σ1R^−/−^) male sigma 1 receptor knockout mice, homozygous (HINT1^−/−^) male HINT1 knockout mice and wild-type (WT) littermate mice were used in the study. The σ1R^−/−^ mice were backcrossed (N10 generation) onto a CD1 albino genetic background (ENVIGO, Milano, Italy). HINT1^−/−^ mice with the genetic background from 129 mice were generously supplied by I.B. Weinstein/J.B. Wang and breeding in our animal facility. The mice used in these experiments were produced from heterozygous breeding pairs and assigned randomly to be used for the different experiments. The genotypes of the WT and KO mice were confirmed by PCR. All mouse housing, breeding and experimental protocols were performed in strict accordance with the guidelines of the European Community for the Care and Use of Laboratory Animals (Council Directive 2010/63/EU) and Spanish law (RD53/2013) regulating animal research. The use of drugs, experimental design and sample size determination were approved by the Ethical Committee for Research of the CSIC (SAF2015–65420 & CAM PROEX 225/14, 1 January 2019). The mice were maintained at 22 °C on a diurnal 12-h light/dark cycle and provided free access to food and water. To reduce the risk of social stress, mice from the same litter were grouped together and remained in these groups throughout the study. The mice were also provided extra space for comfort, as well as nesting material (e.g., soft paper and cardboard refuge) and small pieces of chewable wood. The mice were used when they were between the ages of 6 and 10 weeks. All attempts were made to minimize the number of mice used in each experiment.

The compounds used were as follows—morphine sulfate (Merck, Darmstadt, Germany); NMDA (0114, Tocris). The drugs were dissolved in saline. Doses and intervals were selected based on previous work [46,85]. To facilitate selective and straightforward access to their targets, the compounds were injected (4 μL) into the lateral ventricles of mice as previously described [86]. Animals were lightly anesthetized and injections were performed with a 10 μL Hamilton syringe at a depth of 3 mm at a point of 2 mm lateral and 2 mm caudal from the bregma. The 4 μL were infused at a rate of 1 μL every 5 s. After this the needle was maintained for an additional 10 s. Additionally, in a group of mice the administration of morphine was performed by the intraperitoneal (ip) route. Each group consisted of 6 to 8 animals and mice were randomly assigned to each treatment of the selected compounds.

### 4.2. Formalin-Induced Pain

The formalin test was performed as described with slight modifications [87]. Animals were allowed to habituate to the experimental room for at least 30 min; 20 μL of a 0.5% formalin solution (FO0011005P, Scharlau) or saline as a control, was injected into the plantar surface of the right hind paw. We selected this dose of formalin since it has been previously reported to selectively activate TRPA1 [88]. Immediately after intraplantar injection, the mouse was placed into a glass cylinder and observed. A small mirror was positioned behind the glass cylinder to allow clear observation of the paws. The time spent licking or biting the injected paw for 30 min (divided into six periods of 5 min each) after the injection was measured as an indicator of the pain response. For ex vivo assays, the animals were sacrificed 5 min after the injection of formalin or saline and the ipsilateral and contralateral L3-L4 DRGs and their corresponding spinal cord segments (dorsal T13-L1 sections) were collected.

### 4.3. Chronic Constriction Injury (CCI)

After testing mice for their basal mechanical sensitivity, CCI was performed under isoflurane/oxygen anesthesia [89] using a modification of the Bennett and Xie procedure [90]. Briefly, a 0.5-cm incision was made in the right midthigh, the biceps femoris muscle was separated and the sciatic nerve was exposed proximal to its trifurcation. Two ligatures (5/0 braided silk suture; 70014, Lorca Marin, Murcia, Spain) were tied around this nerve approximately 1 mm apart until a short flick of the ipsilateral hind limb was observed. The incision was then closed in layers with a 4-0 Ethicon silk suture. The tactile pain thresholds of both the ipsilateral and contralateral hind paws were then assessed on different time interval post-surgery. The mice were individually placed in a transparent plastic cage with a wire mesh bottom that allowed full access to the paws. After a habituation period of 20 min, a mechanical stimulus was delivered to the plantar surface from below the floor of the test chamber to measure allodynia using an automatic von Frey apparatus (37450, Ugo Basile, Comerio, Italy). A steel rod (0.5 mm diameter) was pushed against the hind paw over a 10-s period as the force increased from 0 to 10 g. When the mouse withdrew its hind paw, the mechanical stimulus was automatically stopped and the force at which withdrawal occurred was recorded. Animals were sacrificed at the time interval allodynia peaked, which was observed seven days after surgery.

### 4.4. Immunoprecipitation and Western Blotting

The preparation of membrane and the immunoprecipitation assays were performed as described previously [91,92]. The specificity and efficacy of the antibodies used in immunoprecipitation assays have been addressed elsewhere [43,93]. Briefly, the cerebral structures were collected and homogenized in 10 volumes of 25 mM Tris-HCl (pH 7.5) and 0.32 M sucrose supplemented with a 0.2 mM phenylmethylsulphonyl fluoride (PMSF). The homogenate was centrifuged at 1000× *g* for 10 min to remove the nuclear fraction. The supernatant (S1) was centrifuged twice at 20,000× *g* for 20 min to obtain the crude synaptosomal pellet (P2). The final pellet was diluted in Tris buffer supplemented with a 0.2 mM PMSF and a protease inhibitor cocktail (P8340, Sigma, Madrid, Spain), then divided into aliquots and processed for protein determinations. For immunoprecipitation studies, about 800 µg of protein in the P2 pellet was solubilized by sonication at 4 °C (two cycles of 10 s each) in 2 mL volume containing 50 mM Tris-HCl (pH 7.5), 50 mM NaCl, 1% Nonidet P-40, phosphatase inhibitor mixture (P2850, Sigma) and a protease inhibitor cocktail (P8340, Sigma). Solubilization was continued overnight at 4 °C. The lysates were cleared with streptavidin agarose (17-5113-01, GE Healthcare) for 1 h at 4 °C. The solubilized proteins were then incubated overnight at 4 °C with affinity-purified biotinylated IgGs raised against MOR, DOR and NMDAR. The samples were incubated with streptavidin agarose for 2 h and then centrifuged for 5 min at 4300× *g*. The agarose pellets recovered were subjected to five cycles of washing and resuspension in Nonidet P-40 buffer, followed by centrifugation. To detach the immunocomplexes, the samples were heated with 2× Laemmli buffer (1610737, Bio-Rad, Madrid, Spain) for 10 min at 100 °C. The mixture was cooled to room temperature and the streptavidin agarose was separated in a centrifugal filter with a pore size of 0.22 µm (Ultrafree-MC UFC30GV0S, Merck-Millipore, Madrid, Spain). The immunocomplexes were recovered and resolved with SDS-PAGE electrophoresis in 10 cm × 10 cm × 1.5 mm gel slabs (10% total acrylamide concentration, 2.6% bisacrylamide cross-linker concentration). Separated proteins were then transferred onto 0.2 μm polyvinylidene difluoride (PVDF) membranes (162-0176, Bio-Rad) and probed overnight at 6 °C with the selected primary antibodies diluted in Tris-buffered saline pH 7.6 (TBS) + 0.05% Tween 20 (TTBS). Those were detected using secondary antibodies conjugated to horseradish peroxidase. The secondary antibodies were directed to the light IgG chains of the primary antibodies to preserve target immunosignals in the range 40 kDa-60 kDa. In parallel gel blots a fraction of the samples were loaded and bait proteins were detected. The secondary antibodies reacted primarily with the IgG light chains of the primary antibodies and of the accompanying antibodies used for immunoprecipitation. Thus, these signals when needed also provided a loading control for the samples in the gel. The in vitro assays using recombinant proteins did not require immunoprecipitation; thus, IgGs were excluded. To resolve the small proteins, we used 4–12% Bis-Tris gels (NP0341, Invitrogen, Fisher Scientific, Madrid, Spain) with MES SDS running buffer (NP0002, Invitrogen). 

The western blot images and antibody binding were visualized by chemiluminescence (170-5061, Bio-Rad) and recorded using an ImageQuant^TM^ LAS 500 (GE Healthcare). For each blot, the area containing the target protein was typically selected. The software automatically calculates the optimal exposure time for each of the specified areas to provide the highest possible signal to enable accurate quantification of the sample. Protein immunosignals were measured using the area of the strongest signal of each studied group of samples (average optical density of the pixels within the object area/mm^2^; AlphaEase FC software), the grey values of the means were then normalized within the 8 bit/256 grey levels [(256-computed value)/computed value].

The antibodies used for immunoprecipitation assays were directed towards amino acid sequences in the extracellular domains of the membrane receptors. The affinity purified IgGs against the extracellular domains of the MOR second external loop (EL) (205-216: MATTKYRQGSID; GenScript Co., Piscataway, NJ, USA), DOR first EL (110-120: METWPFGELL; GenScript Co.) and the NMDAR NR1 subunit (483-496: KFGTQERVNNSNKK; GenScript Co.) were labeled with biotin following the manufacturer’s instructions (21217; ThermoScientific). The primary antibodies used in Western blotting were as follows: anti-MOR Ct aa 387–398 (GenScript Co.); anti-DOR Nt aa 2-16 (GenScript Co.); anti-NMDAR NR1 (MAB1586, Merck-Millipore); anti-TRPA1 Nt (PAB11992, Abnova, Taipei City, Taiwan); anti-TRPA1 inner sequence (LS-C747641, LSBio, Seattle, WA, USA); anti-σ1R (42-3300, Invitrogen); anti-CaM (05-173, Merck-Millipore, Madrid, Spain); anti-HINT1 aa 93-106 (Inmunostep, Salamanca, Spain).

### 4.5. PNGase F Digestion of Immunoprecipitated Proteins

The NR1 subunit was immunoprecipitated from the solubilized P2 fraction of the mouse spinal cord as described above. The agarose pellets underwent five cycles of washing, followed by centrifugation and resuspension in 1 mL of Nonidet P-40 buffer. At the end of this process, immune complexes were resuspended and solubilized in 100 mM NaH_2_PO_4_ (pH 7.7), 1 mM EDTA, 1% β-mercaptoethanol, 0.5% SDS and 1 mM dithiothreitol and heated at 100 °C for 10 min. The solubilized material was supplemented with 0.65% octylthioglucoside to help remove SDS from the proteins and then incubated with PNGase F (V4831, Promega, Madrid, Spain) for 18 h at 37 °C (5 units/10 μg of protein). The samples were then concentrated, solubilized in Laemmli buffer, separated on a 10% SDS-polyacrylamide gel and blotted. The TRPA1 immunosignals were obtained. 

### 4.6. Recombinant Proteins and In Vitro Interactions between Recombinant Proteins

The coding region of the N- and C-terminal regions of TRPA1 (NP_808449; residues 1–721 and 961–1125, respectively), the C-terminal region of MOR1 (AB047546: residues 286–398), the C0-C1-C2 region of the glutamate NMDAR NR1 subunit (NM_008169: residues 834–938), the full-length murine σ1R (AF004927) and HINT1 (NM_008248), were amplified by RT-PCR using total RNA isolated from the mouse brain as a template. Specific primers containing an upstream Sgf I restriction site and a downstream Pme I restriction site were used as described previously [46,50]. The PCR products were cloned downstream of the glutathione S-transferase (GST)/HaloTag^®^ coding sequence (Flexi^®^ Vector, Promega) and the tobacco etch virus (TEV) protease site and sequencing revealed that the sequences of the proteins were identical to the GenBank™ sequences. The vectors were introduced into *E. coli* BL21 (KRX #L3002, Promega) and clones were selected on solid medium containing ampicillin. After 3 h of induction at room temperature (RT) in the presence of 1 mM isopropyl β-d-1-thiogalactopyranoside (IPTG) and 0.1% rhamnose, cells were collected by centrifugation and maintained at −80 °C. The fusion proteins were purified under native conditions on GStrap FF columns (17-5130-01, GE Healthcare) or with HaloLink Resin (G1915, Promega). When necessary, retained fusion proteins were cleaved on the column with ProTEV protease (V605A, Promega) and further purification was achieved by high-resolution ion exchange (780-0001, Enrich Q, BioRad). Sequences were confirmed by automated capillary sequencing. Recombinant calmodulin (208694, Merck-Millipore) was obtained from commercial sources. 

The N- and C-terminal domains of TRPA1 were immobilized through covalent attachment to N-Hydroxysuccinimide (NHS)-activated Sepharose 4 fast flow (17-0906-01, GE) according to the manufacturer’s instructions. The protein studied (200 nM) was then incubated with either NHS-blocked Sepharose 4FF (negative control) or with the immobilized TRPA1 sequence (100 nM) in 300 μL of a buffer containing 50 mM Tris–HCl (pH 7.5) and 0.2% 3-[(3-cholamidopropyl) dimethylammonio]-1-propanesulfonate (CHAPS) in the presence of 3 mM CaCl_2_. The samples were mixed by rotation for 30 min at RT and the proteins bound to TRPA1-Sepharose 4FF were recovered by centrifugation and washed three times. To study whether the MOR and NR1 subunits interacted with N- or C-terminal domains of TRPA1 via HINT1, σ1R or CaM, the agarose-attached TRPA1–HINT1 complexes were incubated for a further 30 min at RT with rotation in the presence of MOR or NR1 (200 nM) in a reaction volume of 300 μL of 50 mM Tris–HCl (pH 7.5), 3 mM CaCl_2_ and 0.2% CHAPS. This protocol was also carried out to assess if σ1R or CaM could be the link between MOR/NR1 and TRPA1. To facilitate the entrance of CaM into NR1 C1 this interaction was performed as described [46]. Agarose pellets containing the bound proteins were obtained by centrifugation and they were washed thrice in the presence of 3 mM CaCl_2_, solubilized in 2× Laemmli buffer and analyzed by Western blotting.

### 4.7. Statistical Analyses

The Western blot data are expressed as the change in signal relative to that of the control group, which was assigned an arbitrary value of 1. Statistical analyses were performed using the Sigmaplot/SigmaStat v.14 package (Statistical Package For The Social Sciences (SPSS) Science Software, Erkrath, Germany) and *p* < 0.05 was considered to indicate significance. The data were analyzed using one-way ANOVA followed by the pairwise Holm-Sidak multiple comparison test.

## Data Availability

Data is contained within the article or supplementary material.

## Supplementary Materials

The following are available online at https://www.mdpi.com/1422-0067/22/1/229/s1, Figure S1: TRPA1 association with MORs in spinal cord in HINT1-/- and σ1R-/- mice. Figure S2: The HINT1 protein or σ1R does not support the MOR association with the Nt or Ct regions of TRPA1 channels. Figure S3: CaM mediates the TRPA1 Ct association with MOR in the absence of Ca2+. Figure S4: Pharmacological modulation of TRPA1 associations with MORs and glutamate NMDARs. Figure S5: Formalin-induced inflammatory pain alters TRPA1 associations with MORs and NMDARs. Figure S6: TRPA1 associations with opioid receptors and NMDARs in the CCI model of neuropathic pain.

## Abbreviations

CaM	calmodulin
CCI	chronic contrition injury
CNS	central nervous system
DOR	delta-opioid receptor
DRG	dorsal root ganglia
HINT1	histidine triad nucleotide binding protein 1
MOR	mu-opioid receptor
NMDAR	N-methyl-d-aspartate acid glutamate receptor
σ1R	type 1 sigma receptor
TRP	transient receptor potential calcium channel
TRPA1	transient receptor potential ankyrin member 1
WT	wild type
