# Supplementary material for: Calmodulin Supports TRPA1 Channel Association with Opioid Receptors and Glutamate NMDA Receptors in the Nervous Tissue
Source: Int J Mol Sci. 2020 Dec 28;22(1):229. doi: 10.3390/ijms22010229 (PMC7795679; doi:10.3390/ijms22010229)
Supplement: Supplementary file 1 [file ijms-22-00229-s001.pdf]

Supplemental Figures

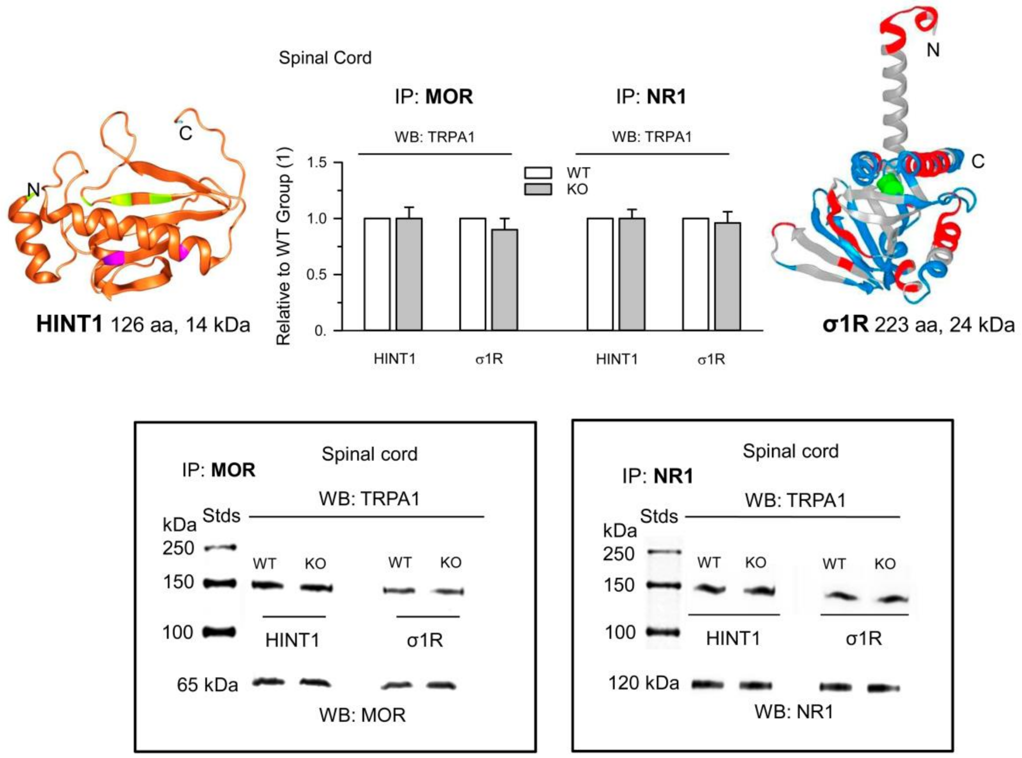

Supplemental Figure 1. TRPA1 association with MORs in spinal cord in HINT1$^{-/-}$ and σ1R$^{-/-}$ mice. The *ex vivo* associations of TRPA1 channels with MORs and NR1 subunits of NMDARs were addressed using the synaptosomal enriched fraction of spinal cord obtained from HINT1$^{-/-}$ and σ1R$^{-/-}$ mice. MORs and NR1 subunits were immunoprecipitated from different solubilized preparations and the presence of TRPA1 proteins was determined by Western blot analysis (see Methods). The bars are the average ± SEM of at least three independent determinations. The control WT group was assigned an arbitrary value of 1, and the data corresponding to the KO group were referred to as the control. Key: IP, immunoprecipitated protein; WB, immunodetected protein by Western blot; WT, wild type control tissue; KO, tissue from the corresponding knockout mice.

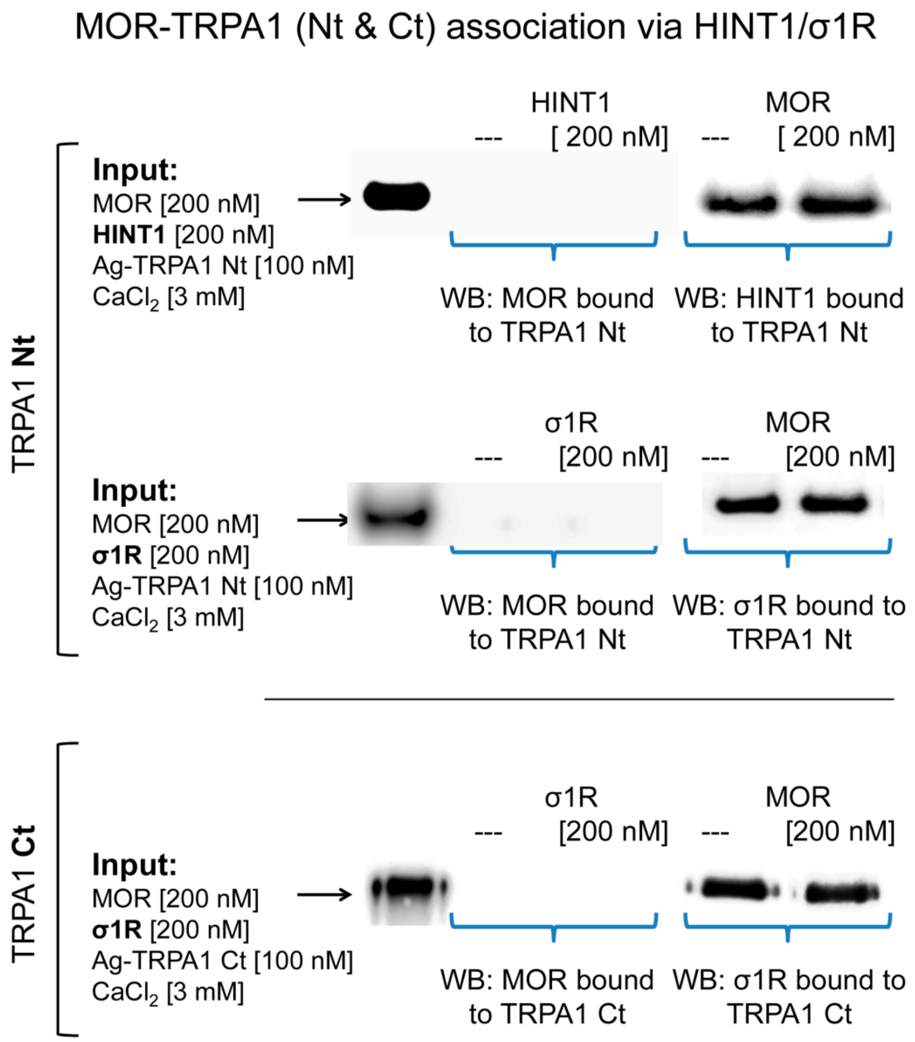

Supplemental Figure 2. The HINT1 protein or σ1R does not support the MOR association with the Nt or Ct regions of TRPA1 channels. The recombinant cytosolic regions of TRPA1 were covalently attached to NHS-agarose beads and then sequentially incubated with MORs and HINT1 proteins or σ1Rs. The assays were conducted in the presence of 3 mM $CaCl_2$. At the end of the incubation, agarose-TRPA1 was recovered by several cycles of washing-resuspension, and the bound proteins were detached with 2x Laemmli buffer and resolved by SDS-PAGE followed by immunodetection. Further details in the Methods. Key: Input, Proteins incubated with agarose TRPA1 in the presence of Ca^{2+}; the MOR protein is immunodetected; WB, immunodetected protein in Western blot.

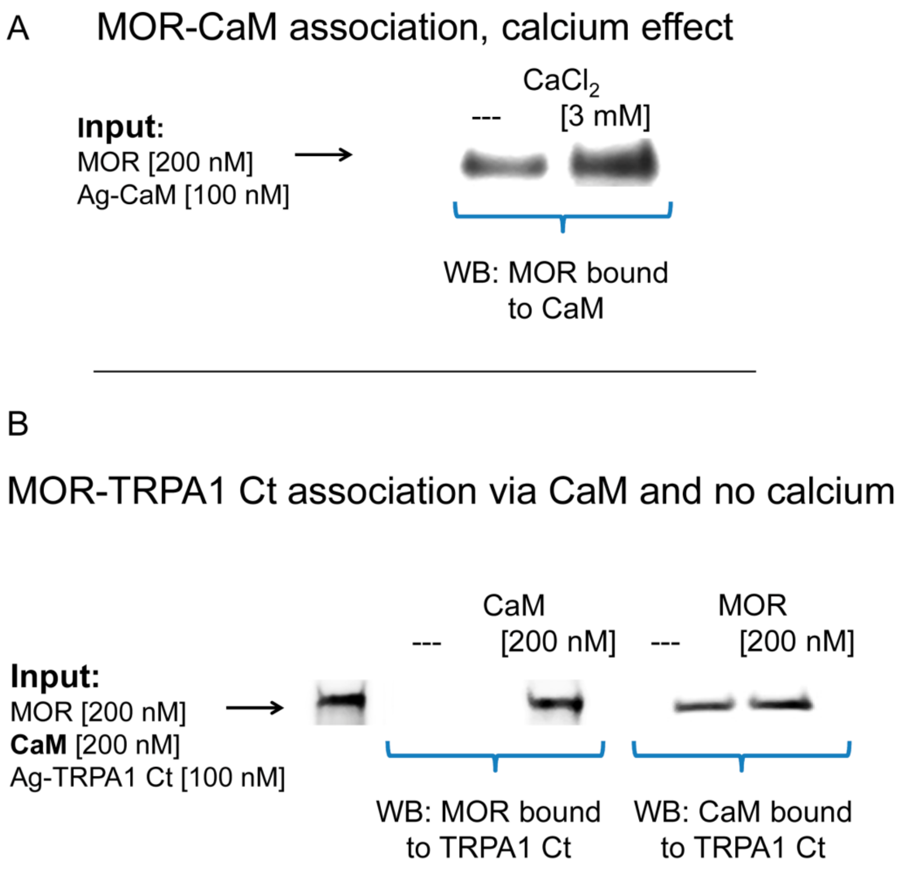

Supplemental Figure 3. CaM mediates the TRPA1 Ct association with MOR in the absence of Ca^{2+}. A, CaM covalently attached to agarose was incubated with MORs in the absence and presence of 3 mM $CaCl_2$. At the end of the incubation, the bound MORs were detached from the agarose-CaM with 2x Laemmli buffer and resolved by SDS-PAGE followed by immunodetection. Further details in the Methods. B, MOR does not associate with TRPA1 in the absence of Ca^{2+}-activated CaM (Fig. 5). To address the role of Ca^{2+} in this association, TRPA1 Ct was coupled to NHS-agarose and incubated with MORs and CaM in the absence of Ca^{2+}. At the end of the assay, MOR and CaM proteins were detached from the agarose-TRPA1 Ct and immunodetected. Details in the Methods. Key: Input, MOR incubated with agarose-CaM or agarose-TRPA1 Ct in the presence of CaM; the MOR protein is immunodetected; WB, immunodetected protein in Western blot.

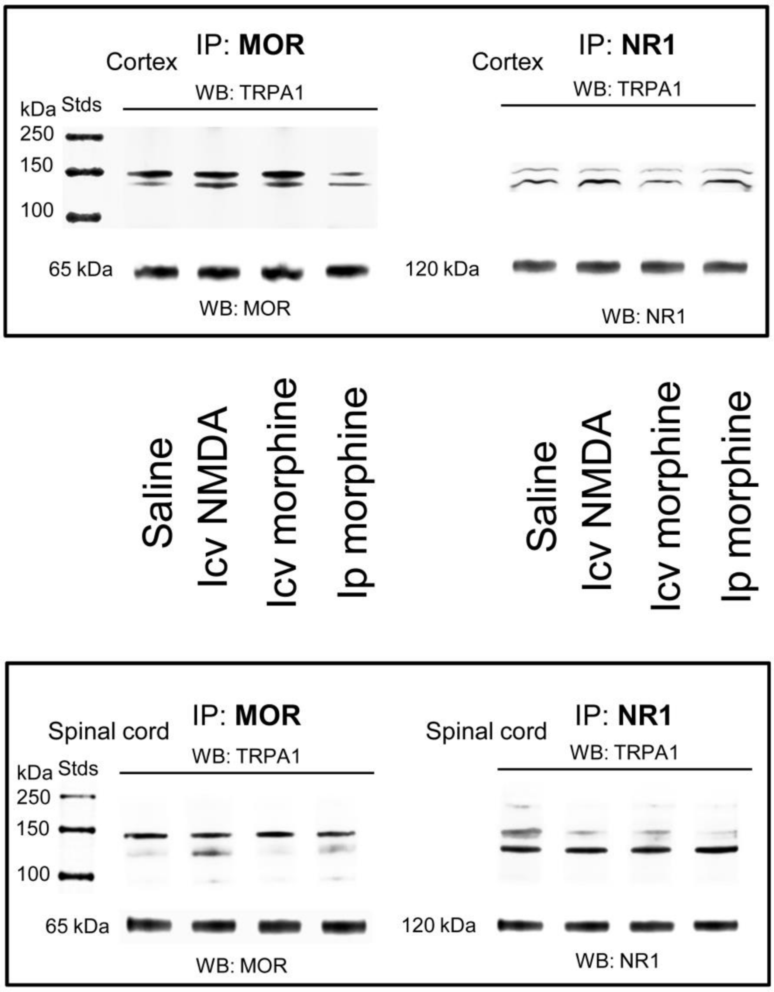

Supplemental Figure 4. Pharmacological modulation of TRPA1 associations with MORs and glutamate NMDARs. The mice were icv-injected with saline, 50 pmol NMDA and 10 nmol morphine. Other group of mice received 10 mg/kg morphine via ip. Thirty minutes after the icv injections, and 60 min after ip morphine injection, the mice were killed and cortical and spinal cord synaptosomal fractions were prepared. Following solubilization of these membrane preparations, MORs and NR1 subunits were immunoprecipitated. The coprecipitated TRPA1 was detached from the bait proteins and analyzed by Western blotting. The assay was typically performed twice or thrice. A representative blot is depicted. Key: IP, immunoprecipitated protein; WB, immunodetected TRPA1 in Western blot; icv, intracerebroventricular injection; ip, intraperitoneal injection; Stds, protein standards, molecular size (kilo Daltons) markers.

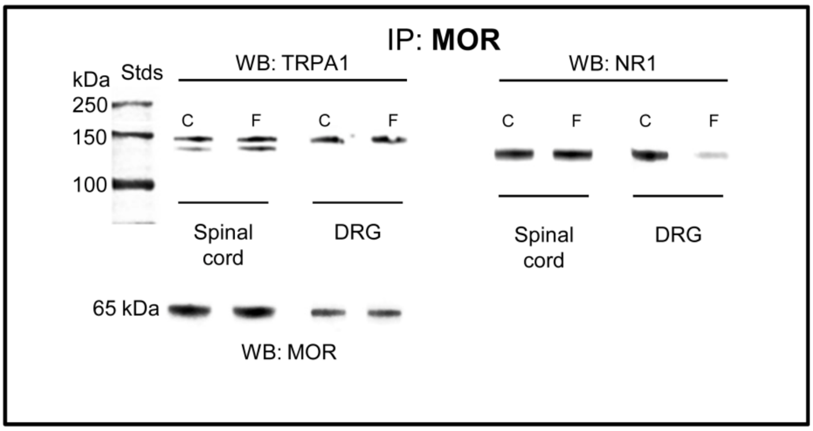

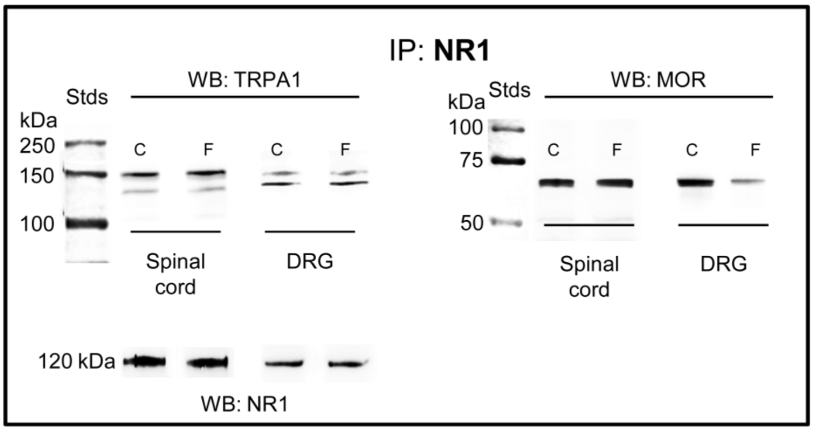

Supplemental Figure 5. Formalin-induced inflammatory pain alters TRPA1 associations with MORs and NMDARs. The mice received an intraplantar injection of saline or 20 μL of a 0.5% formalin solution into the right hind paw. After 5 min the mice were killed and synaptosomal fractions from DRG and spinal cords were obtained. MORs and NR1 subunits were immunoprecipitated from the solubilized membrane preparations. The indicated coprecipitated proteins were then analyzed by Western blotting. Details in the Methods and Supp. Fig. 4. Key: IP, immunoprecipitated protein; WB, immunodetected protein by Western blot; DRG, dorsal root ganglia; C, contralateral to the formalin injection (control group receiving saline); F, ipsilateral to formalin injection.

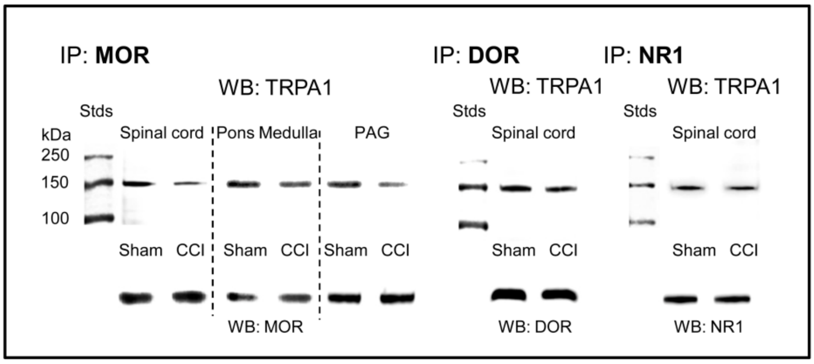

Supplemental Figure 6. TRPA1 associations with opioid receptors and NMDARs in the CCI model of neuropathic pain. Different groups of mice were sham operated or subjected to chronic constriction injury (CCI) of the sciatic nerve. After 7 days the mice were killed and synaptosomal fractions from different neural areas were obtained. The MOR, DOR and NR1 subunits were immunoprecipitated from the solubilized membrane preparations. At the end of the procedure, the coprecipitated TRPA1 proteins were detached from the bait proteins and analyzed by Western blotting. Further details in the Mehods and Supp. Fig. 4. Key: IP, immunoprecipitated protein; WB, immunodetected TRPA1 by Western blotting; PAG, periaqueductal gray matter.
